# An account for barriers and strategies in fulfilling women’s right to quality maternal health care: a qualitative study from rural Tanzania

**DOI:** 10.1186/s12884-018-1990-z

**Published:** 2018-08-30

**Authors:** Thomas Wiswa John, Dickson Ally Mkoka, Gasto Frumence, Isabel Goicolea

**Affiliations:** 1 Health Department, Mkinga District Council, Po. Box 6005 Tanga, Tanzania; 2 Department of Clinical Nursing, School of Nursing, Muhimbili University of Health and Allied Sciences, Dar es Salaam, Tanzania; 3 Department of Development Studies, School of Public Health and Social Sciences, Muhimbili University of Health and Allied Sciences, Dar es Salaam, Tanzania; 4 Department of Public Health and Clinical Medicine, Unit of Epidemiology and Global Health, Umeå University, 901 85 Umeå, Sweden

**Keywords:** Health workers, Health system, Human rights-based approach to health/ right to health approach, Quality, Acceptability, Availability, Accessibility, Services delivery

## Abstract

**Background:**

Tanzania has ratified and abides to legal treaties indicating the obligation of the state to provide essential maternal health care as a basic human right. Nevertheless, the quality of maternal health care is disproportionately low. The current study sets to understand maternal health services’ delivery from the perspective of rural health workers’, and to understand barriers for and better strategies for realization of the right to quality maternal health care.

**Methods:**

Semi-structured in-depth interviews were conducted, involving 11 health workers mainly; medical attendants, enrolled nurses and Assistant Medical Officers from primary health facilities in rural Tanzania. Structured observation complemented data from interviews. Interview data were analyzed using thematic analysis guided by the conceptual framework of the right to health.

**Results:**

Three themes emerged that reflected health workers’ opinion towards the quality of health care services; “It’s hard to respect women’s preferences”, “Striving to fulfill women’s needs with limited resources”, and “Trying to facilitate women’s access to services at the face of transport and cost barriers”.

**Conclusion:**

Health system has left health workers as frustrated right holders, as well as dis-empowered duty bearers. This was due to the unavailability of adequate material and human resources, lack of motivation and lack of supervision, which are essential for provision of quality maternal health care services. Pregnant women, users of health services, appeared to be also left as frustrated right holders, who incurred out-of-pocket costs to pay for services, which were meant to be provided free.

## Background

To date, about two women of reproductive age die every 4 minutes somewhere in the globe due to pregnancy and child birth related complications, most of them in low income countries [1]. In order to avert these deaths the World Health Organization (WHO) recommends, the access to, among other things; family planning services, antenatal care, delivery services, emergency obstetric care (EmOC), and post-natal care [2]. Such services need to be available, affordable and acceptable to all women [3, 4].

Despite the effectiveness of these interventions, in many countries maternal mortality remains unacceptably high, because essential maternal health services are not available as required. This can be considered as a violation of the human rights to health [5, 6]. The universal Declaration of Human Rights, in article 25 addresses that; “motherhood and childhood are entitled to special care and assistance” [7], stipulating the entitlement of health services to all women as right holders, as well as the obligation of the state to provide such services as duty bearers. The article 12 (2), of the *women’s convention* obliges the state to provide essential services to women during pregnancy, childbirth and post-natal period. These rights were further advocated in 1990s by the International Conference on Population and development [8], the Millennium Development Goals (MDGs) and currently, in the Sustainable Development Goals (SDGs) [9].

Tanzania has ratified some legal treaties such as *the Economic Covenant; the Women Convention* and the universal Declaration of Human Rights which emphasizes on the right to health as a fundamental human right [10]. Despite these endorsements, the current maternal mortality rate is still high; 556 per 100,000 live births [11]. Half of expected deliveries in the country takes place at home or other places aside health facility, which deemed them difficult to access essential services in case of emergency [12, 13]. The country is still facing some serious challenges that jeopardize the quality of maternal health services especially in rural areas. Staffing level is between 0.05 to 0.16 per 1000 population which is below the Ministry of health (MoH) recommendation of 2.5 per 1000 population [14], and the available health workers are disproportionately distributed in favor of urban areas [14, 15]. Most of these health workers have increased work load, reduced work morale, and provide sub-optimal quality of health care services which affects their quality and acceptability to users [16–19]. Availability of resources are uncertain, and the accessibility and availability of services is quite unreliable [20].

In combating the challenges, the country through the Ministry of Health, Community Development, Gender, Elderly and Children (MoHCDGEC) set several programs to address them. These includes Primary Health Sector Development Program (PHSDP), which aimed at reducing maternal mortality, to increase coverage of births attended by skilled birth attendants and increase availability of human and material resources [21]. Others includes, The national Road Map Strategic Plan to Improve Reproductive, Maternal, Newborn, Child and Adolescent Health In Tanzania: 2016–2020 (One Plan II) [22] and Health Sector Strategic Plan (HSSP) [23] among others.

### Human right based approach to health framework

The human right based approach to health framework considers availability; accessibility; acceptability and quality of health services as among the key domains to ensure that users can fulfill their right to health care [24]. Availability refers to adequate number and wide coverage of the health facilities in a given area with the capacity to meet the set of standards of health services provision [25]. Acceptability refers to aspects of medical ethics, respect towards cultural norms and values, and sensitivity to age and gender [26]. Cook and Dickens [27] defines accessibility as the access to information, economic and physical access to health care based on non-discrimination, namely there should be available health facilities, which offers interventions of optimal standard, in a respectful way without any form of discrimination. In addition, those interventions need to be accessible without any geographical or financial barrier. Quality of health care refers to the availability of effective and efficient interventions to avert avoidable maternal deaths, such as Emergence Obstetric Care (EmOC) [28, 29].

Health workers are central to the realization of the right to maternal health care. They connect health systems to women, and are accountable to ensure the availability; accessibility; acceptability and quality of these services. To improve the quality of maternal health care services is not, however, the work of service providers alone, this crucial activity need to involve the system as a whole [30]. (Inadequate) quality of care is directly affected by shortage of health personnel, inadequate material resources, insufficiency funds allocated for health and poor governance among other things [31]. According to the right to health framework, health care workers are both duty bearers and right holders, meaning that they should be entitled to a health system that provides them with the information, tools and processes that enable them to offer women the highest standard of maternal health care. However, in many settings this is hardly the case.

The aim of this study was to explore the experiences of health care professionals in delivering maternal health care services in a rural Tanzanian setting. Using the human rights to health framework the study sets to understand the barriers and strategies that health care workers implement in order to ensure that maternal health care services are available; accessible; acceptable; and of good quality.

## Methods

### Study design and setting

This qualitative study was carried out in Mkinga, which is one of the eight districts in the Tanga region of Northern eastern Tanzania. The district is rural, with moderate level of socio-economic development and accessible in terms of transportation and communication. The district has 21 wards, and according to the 2012 Tanzania National Census [32] the population of Mkinga district was 118,065, of these 51% were women. The dominant religions in the area are Muslim and Christian; where Muslims are predominant than Christian. The health care services in the district are largely based on primary public health facilities, with three health centers and 26 dispensaries. At the time, we conducted the study, there was no hospital in the district and none of the facilities provided Comprehensive Emergency Obstetric Care (CEmOC) services. Maternal and child health services including Basic EmOC, were provided from health centers (headed by an Assistant Medical Officer and include; 16-bed inpatient services and minor surgeries and staffed with four clinicians and nine nurses) and dispensaries (clinical officer heads services and they are all outpatient except for deliveries and staffing level here is two clinical officers and two nurses). Patients, who needed caesarean section or blood transfusion, were referred to Tanga Regional Referral Hospital (Bombo hospital) which is approximately 50 to 60 km away from Mkinga district. The government of Tanzania advocates for high coverage of basic EmOC services, (that includes; parenteral antibiotics; oxytocin; parenteral anticonvulsants; manual removal of placenta; removal of product of conception and assisted vaginal delivery) and CEmOC services (that includes Basic EmOC plus blood transfusion and caesarean section) for dispensaries and health centers [23].

### Study participants

Participants in the study were 11 health care workers of Mkinga district who worked at nine facilities (health centers and dispensaries). To ensure maximum variation we included medical attendants (4), enrolled nurses (5) and assistant medical doctors (2). Participants worked in either labor ward and/or antenatal clinic, in nine facilities. Six participants were the only health workers working at labor ward and/ or antenatal clinic at their respective facilities. Two facility managers were also selected due to their involvement in every department, including labor ward and antenatal clinic. Participants differed in terms of sex/gender (3 men and 8 women), and time working at the facility- ranging from 1 year to 29 years. Facilities were located between 30 km and 90 km from the nearest hospital.

### Data collection

TWJ (first author) conducted all the interviews between June and August 2015 that lasted between 60 and 90 min. Participants were visited at their facilities, and requested to choose a convenient time and place for interview. Interviews were conducted in Swahili language by the first author (TWJ), who has a medical background and is a Swahili native speaker. He also has previous experience of working in one of the health centre in Mkinga district. Interviews were conducted using semi-structured interview guide with open-ended questions on issues concerning; availability; accessibility; acceptability and quality of health care and interviewer remained open to other new emerging issues. Information were recorded using notebooks and tape recorders and preliminary analysis of data was done upon collection. The process was useful in generating insight, and emerging issues were followed in subsequent interviews. The sample size was not predetermined, and saturation was attained with 11 interviews, whereas we noticed repetition of the earlier gained information with little or no new information in regards to our research question.

TWJ also did structured observations, whereas he did some observations on arrival at the facility before he conducted interviews, this lasted for about 30 min to 2 h. Things like the appearance of the labor room, the number of health providers at the labor ward and RCH unit, general cleanness of the condition, the presence of electricity, running water, presence of blank patography, sterile gloves, suction machine and oxygen cylinder among others, were noted. The observation took place in seven out of nine facilities.

### Data analysis

Thematic analysis as described by Braun and Clarke [33], was employed and the domains of availability; accessibility; acceptability and quality were used to guide the analysis. Generated data from interviews were reviewed daily to ensure accuracy and completeness. Field notes were checked against audio-recorded information. Recorded interviews were transcribed verbatim and then translated into English. Translation was done in collaboration of two authors who have Swahili language as their mother tongue. Thorough double-check of the translated transcripts against the original was done to ensure quality of the translation. English transcripts were made available to all authors to familiarize with the data and generate insight on the contents.

TWJ in collaboration with other three authors (IG, DAM and GF) conducted the analysis. We used open code version 4 computer software to manage the coding process. Initially codes were developed inductively (inductive approach), these codes were generated by coding every sentence of the transcript. In the second step, codes were examined and re-examined to identify differences and similarities and organized around the conceptual framework domains of availability; accessibility; acceptability and quality as preliminary themes (deductive approach). Other emerging domains beyond the ones included in the framework were identified and labelled accordingly. Triangulation was conducted by combining information from the structured observation of the facilities and the individual interviews. In addition, the researcher’s with different levels of familiarity with the setting and from different disciplines were involved, which can be considered also as triangulation. These strategies enhanced credibility.

We gathered information obtained during the observation, tally them in a sheet, and did simple statistical analysis, and results are presented as frequencies of events as in Table 2.

**Table 1:** Themes and a selection of codes for each one

Selected codes	Themes
male HW take care of women in absence of female HW “I am not going there they are all male HW” male HW hinders women to deliver at the facility A female HW at ANC will attract more women A nurse giving care to three patients at the same time Opting for home delivery due to lack of confidentiality They receive more support from home during delivery We provide less care to our patients	It’s hard to respect women’s preferences
“help them with troubles” “Willingly to work until midnight” Helping them as they come from far away Postponing family issues for patients Staying with patients all times Getting ready to help three patients at a time Taking care of many patients at a time	Striving to fulfill women’s needs with limited knowledge and resources
Going for outreach without payment Providing health services at outreach Advices on the place of delivery Informing them the reason for referral Women lives far from the facility Unable to afford transport expenses	Trying to facilitate women’s access to services at the face of transport and cost barriers

(Note: selected codes are from Health Workers’ perspectives)

(Quotation marks indicates in-vivo codes), *HW* Health Workers, *ANC* Antenatal care

## Results

Three main themes (Table 1) were constructed from the analysis of 11 interviews: ‘It’s hard to respect women’s preferences’, ‘Striving to fulfill women’s needs with limited knowledge and resources’ and ‘Trying to facilitate women’s access to services at the face of transport and cost barriers’. These themes described barriers/ challenges experienced and strategies employed by health workers to fulfil women’s rights to maternal health.

**Table 2:** Proportion of the presence of clinical instruments/tools or recommended drugs observed in seven out of 9 visited facilities, *n* = 7

No	Observed physical objects	N
1.	Reliable source of light	Available in 5 out of 7 facilities
2.	Transport for obstetric referral	Available in 2 out of 7 facilities
3.	Suction machine+adult tubes	Available in 5 out of 7 facilities
4.	Suction machine+children tubes	Available in 6 out of 7 facilities
5.	Examination glove (sterile)	Available in 2 out of 7 facilities
6.	Ringer’s lactate	Available in 5 out of 7 facilities
7.	Any antibiotic injection	Available in 5 out of 7 facilities
8.	Oxytocin injection	Available in 4 out of 7 facilities
9.	Clean running water	Available in 5 out of 7 facilities
10.	Intravenous cannula – size 16, 18 or 20	Available in 5 out of 7 facilities
11.	Normal saline	Available in 5 out of 7 facilities
12.	Blank partograph	Available in 5 out of 7 facilities
13.	Cord ligatures	Available in 3 out of 7 facilities
14.	Oxygen cylinder/ Oxygen concentration machine	Available in 1 out of 7 facilities
15.	Magnesium sulphate injection	Available in 4 out of 7 facilities

### It’s hard to respect women’s preferences

Participants pointed out that many women chose to deliver at home over the health facility partly due to perceived lack of emotional support. They explained how women preferred to give birth with traditional birth attendants at home, because they provided such support, e.g. they were patted well and told to sleep to wait for the baby to come down. At home, relatives were allowed to be with the women before, during and after delivery, which made women to feel secured. At health facilities, relatives were not allowed.

Participants also perceived that the health facility setting lacked respect to their clients. This was considered the reason that a woman might deliver her first child at the facility, but not the second and other subsequent children.

*“…, but nurses also might contribute, because you might find out for a woman giving birth of her second pregnancy at home, it could be due to reason that she didn’t like our services when she delivered her first child….at home they say they receive good care, even their relatives are allowed to be with her, but here at facility she would be alone, and one nurse taking care of many patients ….so they feel like they are not provided enough care” [Participant 10]*

Lack of confidentiality was also perceived to influence women to give birth at home. This was the case for HIV positive patients, who preferred to give birth at home due to the fear of discloser HIV status.

An important issue that participants highlighted was that women did not accept male health care providers during delivery, antenatal care or postnatal care services. Participants mentioned some of the reasons for non-acceptance of male providers; whereas religious beliefs and families’ influences played a big role especially during delivery.

*“… we have that one too [that male health providers are not preferred for delivery] , and here it is in it’s extreme, and they say it’s due to religious beliefs, … that a woman should be served by her fellow woman, …recently I was just walking around and I overheard people [males] saying that these male health providers are just looking at our wives genitals, and another one said, they also put their fingers into their private parts, ….but it depends on women to women, some of them are willingly to be delivered by male and others are not”. [Participant 9]*

Participants acknowledged that it was not possible to ensure the availability of female health workers all the times, and described how they tried to educate women to accept male health workers, with different success.

*“I tell women that whenever I am not around, they should get used to male care providers, they are also health care providers like me. Because sometimes I might be, sick or have some family matters I have to attend to. …. For those who understand they just take it easy, but for others it is difficult to accept easily. They will tell you “I am not going there; they are all males””. [Participant 1]*

In instances like these [Shortage of health workers], our respondents expressed frustrating working environments, which hindered their ability to provide quality and respectful maternal health care.

*I have had such cases, I was taking care of three patients at the same time, I placed one of them in the labour ward, another one in the room behind this, and the third in the resting room, then I stayed at the middle, after I had every equipment needed for each patient in place, … If one of them calls, I just put on gloves, and help one after another…. I have encountered situations like these several times…And I am forced to serve them, because they have come to me, for better satisfaction …. So, I will run about with them, and God helps. [Participant 1]*

### Striving to fulfill women’s needs with limited resources and knowledge

The infrastructure of the health care facilities visited was inadequate. Some facilities did not have running water, electricity, and other necessary equipment as shown in Table 2 below. These deficits put quality of health in jeopardy, for example, in facilities without electricity, charcoal was used as source of energy to sterilize their equipment and hurricane lamps as the source of light. In some instances, they had to use and hold up their torches by mouth when conducting deliveries.

Participants pointed out that some services were good, but medications and equipment were not constantly available (as evidenced in Table 2 above). They acknowledged receiving untimely and inadequate medications and equipment from Medical Store Department (MSD) that could not meet their clients’ demands. Health workers perceived the impact of the poor availability of drugs on the quality of care provided to women.

*“… [When] we do not have delivery equipment, for example, gloves… We find it difficult to help poor women… , they [women] don’t have anything, even gloves …I mean most of them come with nothing, not mackintosh (plastic mattress) or gloves, cord cramp or syringe, though all those [equipment] were supposed to be available here but it’s unfortunate we don’t have”. [Participant 10]*

Moreover, health workers perceived availability of equipment and medications as a way to attract women to facilities. In connection to the need of more equipment and conducive working conditions, health workers also saw the need for more health workers, in order to reduce waiting times and enhance quality.

This lack of resources frustrated health care professionals, but also made them to actively try local strategies to remedy theses shortfalls, since as one participant put it: *“women don’t deserve problems; they need to find everything readily available”* [Participant 3]. Locally, they might exchange drugs and equipment with their neighboring facilities.

At the district level, various efforts were mentioned, such as the use of user fee collections and returns from the National Health Insurance Fund (NHIF) and Community Health Fund (CHF) to enable them to buy medications aside medical store department (MSD), although the amount collected did not suffice.

*“…. when we get the money from CHF... we sit down and plan on what to buy. We only buy important things that we do not have … things like sterilizing solutions, gloves etc... Because it is not a lot of money, we just buy few supplies and we use them for short time”. [Participant 8]*

Health workers experienced hard situations and heavy workload in their day-to-day activities. Due to limited staff, existing health workers spent their extra time with patients instead of their families, and at times, they had to skip vacations and annual leaves, as they felt obliged to help women whenever they come at the facility. They also described how they sometimes even helped their clients financially to facilitate their transportation in case of referral.

*“… we usually help them [in transport] ...we give them money ...but we often tell them to prepare themselves whenever they come to clinic, and when they come for delivery we usually ask them if they have money for transport. If they don’t have, we just contribute from among of us and transport them”. [Participant 3]*

Health providers also explained how inadequately involved they felt in their career development especially on issues concerning maternal health. Only few reported to have attended trainings like; Basic EmOC, Focused Antenatal Care, or family planning, and they complained that seminars attendances were unevenly distributed.

This fact, of insufficient knowledge explains the feelings of disempowerment among health care providers. They were depending on the directives and orientations provided during supportive supervision, which were deemed insufficient.

*“… When they come for supervision, they find you working, when would they teach you and make you understand? I think these are just politics, you can’t be taught during supervision, they come when you have a lot of patient.... I can’t understand when they teach me, because teaching somebody needs time”. [Participant 5]*

### Trying to facilitate women’s access to services at the face of transport and cost barriers

Participants further noted on the distance between the facility and where their clients live, and described that in some areas the closest distance from the community to the health facility was 16 km away. Things were even worse in some settings due to the lack of reliable transport from women’s villages to facilities. Motorcycles were the only available transports that were deemed not to be convenient for pregnant women, especially when in labor.

*“It’s true, there is a distance …others might tell you: “I was on my way to come [to facility], but I gave birth by the road”. …. but we also tell them that they should always start their way to come, when labor has just started.... there are a lot of motorcycles now-a-days …but still the geography of this place is not good. …. but they try to come to clinic”. [Participant 3]*

Health workers also felt disempowered to facilitate referrals. This was the case for facilities, which lacked ambulances; on such circumstances they had to face difficulties to refer patients from primary to higher-level facilities. Moreover, for facilities, which had ambulance in most of cases, fuel was not available or not enough, and patients were required to pay for the fuel. Participants explained that women were required to contribute up to Tzs 40,000/= around (20USD) to cover the fuel cost in which most of them could not afford.

*“...We have transport [ambulance], but it has its challenges too…. We don’t have enough fuel …., so patients are supposed to pay for fuel. They don’t like it, but what else can we do, they feel like we are denying them their rights”. [Participant 7]*

Health workers found outreach activities to be useful as it enabled them to provide services to their clients in remote settings. Even if scarce financial support from the district authority seemed to hinder those efforts, health workers would keep trying to provide at least antenatal care services.

*“…This time we are not paid for outreach, I just decide to go...We usually go to nearby villages, especially for RCH, I usually go and do clinics and give them medications [hematinic] and other things needed during that time, I also advise their husbands to make sure their women take medication”. [Participant 1]*

Outreach works were considered useful for antenatal and postpartum care but not delivery although at times health workers had go to provide delivery services to patients who could not be transported to the facilities.

## Discussion

This study has explored experiences of health care professionals in delivering maternal health care services in a rural Tanzanian setting. Using human right to health approach, the study set to understand the limitations that health care workers face and the efforts they undergo to ensure that the services they provide are available; accessible; acceptable and of good quality. Despite the poor working condition, [which is the case for many rural facilities in low resource settings] health workers were enthusiastic about their jobs and tried diverse remedies to improve the services they provided. Strategies like; working for long hours, skipping vacation and borrowing medication and equipment from other facilities, partly assured the availability of equipment in their respective facilities. Women were also made responsible for their own health care, in which they had to buy medication and equipment as well as to pay for their transportation to referral sites.

### Erratic availability of material and human resource

Health workers are the bridging link between health system and the community, the ultimate responsible for fulfilling women’s rights to maternal health care. In order to be able to fulfill that role, health professionals require good working condition, availability of supplies, and reliable referral system among others. Albeit the reality is different, Mkoka et al. [34], documented the poor working condition in other Tanzanian contexts, whereas lack of electricity and other reliable source of light, put health workers, mothers or babies at the risk of infection through cross contamination, and risks to some injuries from sharp objects used during delivery. Our study and some other studies [17, 18, 34–37] from Tanzania reported similar findings.

The right to health approach demands, the state to be accountable for people’s health, by investing in health, equipping facilities with adequate human and material resources that will enable the facility to provide standard health care [7, 38]. The current study has found erratic availability of equipment and supplies.

### Frustrated right holders and disempowered duty bearers

In the present study, health workers reported considerable variation of knowledge and skills regarding maternal health care provision. Improvement of clinical skills of health workers, training, continuous education, motivation and support are considered central to such capacity building [39, 40], but little is being done in many places [14, 34, 41]. The human rights approach [42], requires the state to ensure, its health workers obtain the required knowledge and skills, and offers continuous education to catch up with the advancement treatment and technology [39, 40].

Apart from being provided with satisfactory working condition, health care professionals need to be motivated and supplied with other benefits to enable them to fulfill their duties [41, 43]. All of the studied facilities reported shortage of health workers, mainly nurses. In such rural settings challenges of shortage and failure to retain health workers has been their long lived problem [40]. Availability of adequate health workers, reduces waiting time, increases efficiency of work, and importantly attracts women to facility [44]. By being inadequately empowered with skills and knowledge, less motivation and insufficiently supplied with resources, the present study describes health workers as *frustrated right holders and disempowered duty bearers*. Frustrated right holders, refers to health workers’ whose working conditions and (lack of) resources do not facilitate their duties and rights. Disempowered duty bearers refers to inadequate empowerment and resources to fulfill their day to day obligations of providing services to their clients [45]. The present study, explicitly describes work overload, limited number of health care providers, long working hours and unavailability of drugs and equipment for the provision of health care. These facts reflect denied rights and disempowerment of health workers.

There are dedicated health workers around the globe, who show some commitment far beyond their duties [39]. That is, despite the circumstances of unavailability of resources, they try to do whatever is necessary to rescue their clients. In this study, we highlighted similar commitments used by health workers as strategies to fulfil their obligations of providing services in such difficult conditions. In case of unavailability of equipment and supplies, health workers used returns from health insurances such as CHF and NHIF to buy essential equipment and drugs. However, these returns deemed not enough to afford demands and pregnant women who are entitled to free health care services [46], were made responsible for their health. In this instance, they bought equipment and medication for themselves. This is regarded as a normal practice, and it is also reported elsewhere [47, 48], to mitigate the unavailability of equipment and supplies required for delivery. In such circumstances therefore, health system leave women, especially those in the poorest groups, as *frustrated rights holders,* whose right to available quality health care is denied.

### Disrespectful health care services limit acceptability

Our results also revealed several aspects of (in)-acceptability of health care services, embedded with cultural, religious and contextual factors, i.e. non-acceptance of male providers. To the best of our knowledge, the magnitude of acceptability of male health care providers in childbirth services has not received much attention.

The literature describes disrespect and violent behavior of health workers [48, 49]. Even if our study was based on professionals’ voices, disrespectful practices of professionals were mentioned. Like in other studies [48, 50], the interviewed health workers questioned if their services were really respectful. Negative attitudes of health workers towards patients exist. Mahiti et al. [51] and other studies [16, 52–54] report health worker’ rude language towards their clients, negligence and use of alcohol during working hours. The magnitude of disrespectful and/ or abusive treatment is estimated to be between 19 and 28% of women at health care post [54].

Acceptability of health service, is subject to reputation of the quality of the health services provided as well as disrespectful experiences encountered at last time they used the facility [48, 49] The aspects of acceptability of health care services need to be well understood to enhance a patient-centered care and to fulfil their right to health.

### Ineffective coverage

Health system has to ensure equity in access to health care, in such a way that, those who cannot afford are covered [25, 31]. There is considerable variation of coverage of health care facilities in Tanzania, in which geographical barriers, distance and lack of reliable transport are major challenges [50, 55, 56]. This situation was also found in the present study which describes disempowerment of health care provided and denial of women’s right to health. And in most cases, those residing in remote areas are the most underserved and affected population [50, 57, 58]. Our health workers showed commitment regarding to access of health care, they conducted some outreach activities, even without being compensated.

### Study limitations

We explored health workers’ perspectives, trying to understand how services are delivered to users. We acknowledge that, quality can best be judged by women or other people of the community as highlighted in another study [59]. In future studies, it will be interesting to capture the users’ perspective and contrast it with that of the professionals. Nevertheless, it is also important to know how health workers perceive quality and barriers for facility utilization, as they are also members of the community in which their clients live, and play a key role in supporting women’ s exercise of their right to maternal health care. In our findings, we reported women’s information from health workers’ perspectives, in which their views might be less critical than those from women did. The interviewed health workers were the ones who were found at the facility, who were probably motivated and might put health workers in a better position. Eleven interviews were conducted, which might seem a small sample. However, during the last interviews, the same issues emerged repeatedly, and the variety of informants allowed us to gather information from diverse perspectives. This study triangulated method by in-depth interviews and structured observation to enhance credibility. Interviews were conducted in Swahili and then translated to English; we acknowledge that some of the meanings of the informant might have been lost through the translation process. However, three authors with Swahili language as their mother tongue were involved in translation process.

## Conclusion

Health system presents a gap in provision of quality maternal health care, due to unavailability; inaccessibility and unacceptability of resources. Health workers link health system and community, and they are central to provision of quality maternal health care services, but they are left as *frustrated right holders and disempowered duty bearers*. Their rights such as, adequate training, supervision, motivation and conducive working environment needs to be fulfilled to enable them to fulfill their obligations of providing quality maternal health services. Availability of adequate human and material resources will not only enable health workers to deliver quality health services, but will also help women who are *frustrated right holders* to realize their rights to health care, despite their socio-economic status.

## Abbreviation

A.M.O: Assistance Medical Officer
ANC: Antenatal care
BEmOC: Basic Emergency Obstetric Care
CEmOC: Comprehensive Emergency Obstetric Care
CHF: Community Health Insurance
EmOC: Emergency Obstetric Care
HIV: Human Immunodeficiency virus
HSSP: Health Sector Strategic Plan
IDI: In-depth interviews
MoH: Ministry of health
MoHCDGEC: Ministry of Health, Community Development, Gender, Elderly and Children
MSD: Medical Store Department
NHIF: National Health Insurance Fund
NIMR: National Institute of Medical Research
OPD: Out patient department
PNC: Postnatal care
PPH: Post-partum Hemorrhage
PSDP: Primary Services Development Program
RCH: Reproductive and Child Health
WHO: World Health Organization
